# Imaging classification of prostate cancer with extracapsular extension and its impact on positive surgical margins after laparoscopic radical prostatectomy

**DOI:** 10.3389/fonc.2024.1344050

**Published:** 2024-03-06

**Authors:** Jun-Guang Wang, Chao Zhong, Ke-Cheng Zhang, Jun-Bo Chen

**Affiliations:** Department of Radiology, Ningbo Yinzhou No. 2 Hospital, Ningbo, Zhejiang, China

**Keywords:** prostate cancer, positive surgical margins, magnetic resonance imaging, extracapsular extension, biopsy grade group

## Abstract

**Abstract:**

To explore the impact of different imaging classifications of prostate cancer (PCa) with extracapsular extension (EPE) on positive surgical margins (PSM) after laparoscopic radical prostatectomy.

**Methods:**

Clinical data were collected for 114 patients with stage PT3a PCa admitted to Ningbo Yinzhou No. 2 Hospital from September 2019 to August 2023. Radiologists classified the EPE imaging of PCa into Type I, Type II, and Type III. A chi-square test or t-test was employed to analyze the factors related to PSM. Multivariate regression analysis was conducted to determine the factors associated with PSM. Receiver operating characteristic curve analysis was used to calculate the area under the curve and evaluate the diagnostic performance of our model. Clinical decision curve analysis was performed to assess the clinical net benefit of EPE imaging classification, biopsy grade group (GG), and combined model.

**Results:**

Among the 114 patients, 58 had PSM, and 56 had negative surgical margins. Multivariate analysis showed that EPE imaging classification and biopsy GG were risk factors for PSM after laparoscopic radical prostatectomy. The areas under the curve for EPE imaging classification and biopsy GG were 0.677 and 0.712, respectively. The difference in predicting PSM between EPE imaging classification and biopsy GG was not statistically significant (P>0.05). However, when used in combination, the diagnostic efficiency significantly improved, with an increase in the area under the curve to 0.795 (P<0.05). The clinical decision curve analysis revealed that the clinical net benefit of the combined model was significantly higher than that of EPE imaging classification and biopsy GG.

**Conclusions:**

EPE imaging classification and biopsy GG were associated with PSM after laparoscopic radical prostatectomy, and their combination can significantly improve the accuracy of predicting PSM.

## 1 Introduction

An extracapsular extension (EPE) of prostate cancer (PCa) is a locally advanced PCa, for which patients can choose comprehensive treatment with surgery as the initial treatment. The incidence of EPE has significantly decreased with the promotion of prostate-specific antigen (PSA) screening, but 31% of patients still exhibit pathological evidence of EPE after radical prostatectomy (RP) (1). Positive surgical margins (PSM) are one of the significant adverse pathological findings after RP and are associated with biochemical recurrence (BCR) and disease progression (2, 3). PSM are predictive factors for BCR of PCa (4–7), with a 5-year BCR of up to 40.7% in patients with PSM, whereas patients with negative surgical margins (NSM) have a 15.1% BCR (8). Compared to men with NSM, those with PSM have a significantly higher risk of death due to PCa (9, 10). Among patients undergoing RP, 20-30% have PSM (11); the risk of PSM increases significantly in patients with PCa along with EPE (12).

Imaging examination plays an important role in the diagnosis and treatment of PCa. The latest research shows that Micro-ultrasound (MUS) has been proposed for the diagnosis and staging of PCa, and has a very high sensitivity. As part of the new imaging exams, prostate-specific membrane antigen positron emission tomography/CT (PSMA PET/CT) has gained great popularity in recent years, it is currently recommended in selected patient with BCR after curative treatment for PCa (13). Multiparameter magnetic resonance imaging (mp-MRI) is one of the important approaches for preoperative evaluation of EPE, and can clearly display the anatomical structures of the pelvic cavity and determine the location and extent of EPE (14). Mp-MRI is the most suitable imaging tool for evaluating the staging of PCa (15). EPE on MRI is considered a risk factor for PSM after laparoscopic radical prostatectomy (LRP) (16). However, the relationship between EPE on MRI and PSM is still debatable. In addition, no imaging classification of EPE has been reported for predicting PSM. In this study, we classified the imaging of EPE into Type I, II, and III based on the location of EPE, and discussed the impact of different imaging classifications of EPE on PSM post-LRP.

## 2 Materials and methods

### 2.1 Study population

A total of 680 cases of LRP were collected from September 2019 to September 2023 at Ningbo Yinzhou No. 2 Hospital. Among them, 157 cases (23%) were pathologically confirmed as PT3a stage after surgery.

Exclusion criteria consisted of no preoperative MRI assessment(n=9), insufficient prostate biopsy data(n=13), neoadjuvant endocrine therapy prior to prostate surgery(n=10), and incomplete clinical data(n=11). Finally, 114 patients were included.

### 2.2 Multiparameter magnetic resonance imaging

All patients underwent MRI within 3 months prior to radical surgery using a 1.5T MRI scanner (GE SIGNA Voyager). The scanning sequences included high-resolution T2-weighted imaging (TR4500 ms, TE110 ms), T1-weighted imaging (TR541 ms, TE15 ms), and perfusion-weighted imaging (TR6900 ms, TE100 ms, b=1500 s/mm^2^). Then, dynamic contrast-enhanced T1WI (TR4.20 s, TE1.70 ms) imaging was performed with 15 acquisitions, each lasting for 11 s. The field of view was 24 x 24 cm, and the slice thickness was 3 mm. The image was retrospectively analyzed by a senior radiologist to review the preoperative MRI data of the patients. Based on the imaging manifestations of EPE of PCa, they were classified into three types: Type I, tumor EPE located within the range of 2 to 4 of a clock and 8 to 10 of a clock; Type II, tumor EPE located within the range of 4 to 8 of a clock; Type III, tumor EPE located within the range of 10 to 2 o’clock, including the capsule.

### 2.3 Prostate biopsy

For prostate biopsy, all patients underwent standard systematic transperineal biopsy (12 cores) and an additional 1-3 cores were obtained for lesion identification on MRI. EPE was defined as cancer cells crossing the prostatic capsule into the surrounding adipose tissue.

### 2.4 Extracapsular extensions and positive surgical margins

To determine the location of EPE, the gross pathology report was analyzed, and it was classified into Type I, Type II, and Type III using the same classification system as the EPE imaging classification. PSM was defined as tumor cells on the inked surface of the prostate specimen. All patients underwent laparoscopic radical prostatectomy under general anesthesia.

### 2.5 The variables

Clinical variables included age, body mass index (BMI), prostate volume (PV), preoperative PSA, the International Society of Urological Pathology (ISUP) prostate biopsy grade group (GG) score (Gleason scores ≤ 6, 3 + 4, 4 + 3, 8, and 9-10 corresponding to GG 1-5), percentage of positive cores, operative time, and intraoperative blood loss. MRI variables included the imaging classification of EPE.

### 2.6 Statistical analysis

All statistical analyses were performed using SPSS (v17.0), MedCalc (V20.0), and Stata (V17.0) software. Comparisons between variables were performed using chi-square tests or independent sample t-tests. Unless otherwise specified, data are presented as medians with interquartile ranges (IQRs). The independent risk factors for PSM were determined by multivariable logistic regression analysis, and receiver operating characteristic (ROC) curves were plotted for each risk factor and combination of risk factors. The area under the curve (AUC) was calculated, and differences were compared using the DeLong test. A P-value <0.05 was considered statistically significant. To plot the clinical benefit of predicting positive surgical margins for each risk factor and combination of risk factors, a decision curve analysis was performed. Finally, a probability estimation table based on the risk factors was constructed for PSM.

## 3 Results

### 3.1 Descriptive statistics

Themedian (IQR) age of 73 (69-76) years, BMI of 23.7 (21.2-25.6) kg/m^2^, PSA level of 15.3 (8.9-37.8) ng/ml, PV of 33.7 (24.8-43.1) ml, percentage of positive biopsy cores of 50 (33-75)%, surgical time of 160 (120-204) min, and intraoperative blood loss of 100 (50-150) ml. The biopsy of patients in GG of 8, 20, 25, 33, and 28 were 1, 2, 3, 4, and 5 respectively (**Table 1**).

**Table 1:** Patients’ characteristics (n = 114).

		Median	IQR
Age	years	73	69-76
BMI	kg/m^2^	23.7	21.2-25.6
PSA	ng/ml	15.3	8.9-37.8
PV	ml	33.7	24.8-43.1
Biopsy GG, n(%)	1	8	(4)
	2	20	(10)
	3	25	(13)
	4	33	(17)
	5	28	(14)
Percent positive cores	%	50	33-75
Operative time	min	160	120-204
Intraoperative blood loss	ml	100	50-150

BMI, body mass index; GG, grade group; IQR, interquartile range; PSA, prostate specific-antigen; PV, prostate volume.

### 3.2 Clinicopathological and multiparameter magnetic resonance imaging factors associated with positive surgical margins

The PSA levels were significantly higher in patients with PSM compared to those with NSM (20.9 [11.4-43.9] vs. 11.3 [7.1-26.8] ng/mL, p<0.05). The percentage of positive cores was also higher in patients with PSM than in those with NSM (60 [42-82] vs. 425 [26-73]%, p<0.05). There were significant differences between patients with PSM and NSM in biopsy GG (p<0.05) (**Table 2**). According to the imaging classification of EPE in PCa, 14 cases of the 114 cases (12.4%) were of Type I, 50 cases (43.8%) of Type II, and 50 cases (43.8%) of Type III. The PSM for Types I, II, and III were 14.2%, 44.0%, and 68.0% respectively, with statistically significant differences among the groups (p<0.05) (**Table 2**).

**Table 2:** Comparison of clinicopthological and mp-MRI factor.

	positive surgical margin (n=58)	negative surgical margin (n=56)	P- value
Clinicopathological			
Age, years	74 (68-76)	73 (70-76)	0.870
BMI, kg/m^2^	23.7 (21.9-25.7)	23.5 (20.8-25.3)	0.805
PSA, ng/ml	20.9 (11.4-43.9)	11.3 (7.1-26.8)	<0.05
PV, ml	33.7 (24.4-43.8)	33.7 (25.5-42.7)	0.542
Biopsy GG, n(%)			<0.05
1-2	6	22	
3-4	30	28	
5	22	6	
Percent positive cores, %	60 (42-82)	42 (26-73)	<0.05
Operative time, min	175 (145-205)	150 (120-200)	0.174
ntraoperative blood loss, ml	100 (50-150)	70 (50-120)	0.078
Mp-MRI			
Imaging classification of EPE, n(%)			<0.05
Type I	2 (3)	12 (21)	
Type II	22 (38)	28 (50)	
Type III	34 (59)	16 (29)	

Data are shown by median (IQR) unless otherwise indicated. BMI, body mass index; EPE, extraprostatic extension; GG, grade group; IQR, interquartile range; MRI, magnetic resonance imaging; PSA, prostate-specific antigen; PV, prostate volume.

### 3.3 Multivariate analysis of positive surgical margins

Thebiopsy GG (GG3-4: odds ratio [OR] 0.298, 95% confidence interval [CI] 0.104-0.858, p<0.05; GG5: OR 6.021, 95% CI 1.253-28.940, p<0.05) and EPE imaging classification (Type II: OR 16.357, 95% CI 1.433-86.647, p<0.05; Type III: OR 35.901, 95% CI 3.139-199.007, p<0.05) were found to be risk factors for PSM (**Table 3**).

**Table 3:** Multivariate analysis for predicting positivesurgical margin using clinical and MRI parameters (n = 114).

Variables	N	OR	95%CI	P-value
Age, years	114	0.958	0.877-1.046	0.339
BMI, kg/m^2^	114	1.059	0.912-1.230	0.451
PSA, ng/ml	114	1.016	0.992-1.041	0.182
PV, ml	114	0.986	0.958-1.015	0.342
Biopsy GG, n(%)				
1-2	28	ref.	ref.	ref.
3-4	58	0.298	0.104-0.858	<0.05
5	28	6.021	1.253-28.940	<0.05
Percent positive cores, %	114	0.838	0.089-7.909	0.877
Operative time, min	114	1.001	0.992-1.010	0.802
ntraoperative blood loss, ml	114	1.007	0.998-1.015	0.119
Imaging classification of EPE, n(%)				
Type I	14	ref.	ref.	ref.
Type II	50	16.357	1.433-86.647	<0.05
Type III	50	35.901	3.139-199.007	<0.05

BMI, body mass index; CI, confidence interval; EPE, extraprostatic extension; GG, grade group; OR, odds ratio; PV, prostate volume; PSA, prostate-specific antigen; ref, reference.

### 3.4 Receiver operating characteristic analysis for positive surgical margins

**Figure 1:**
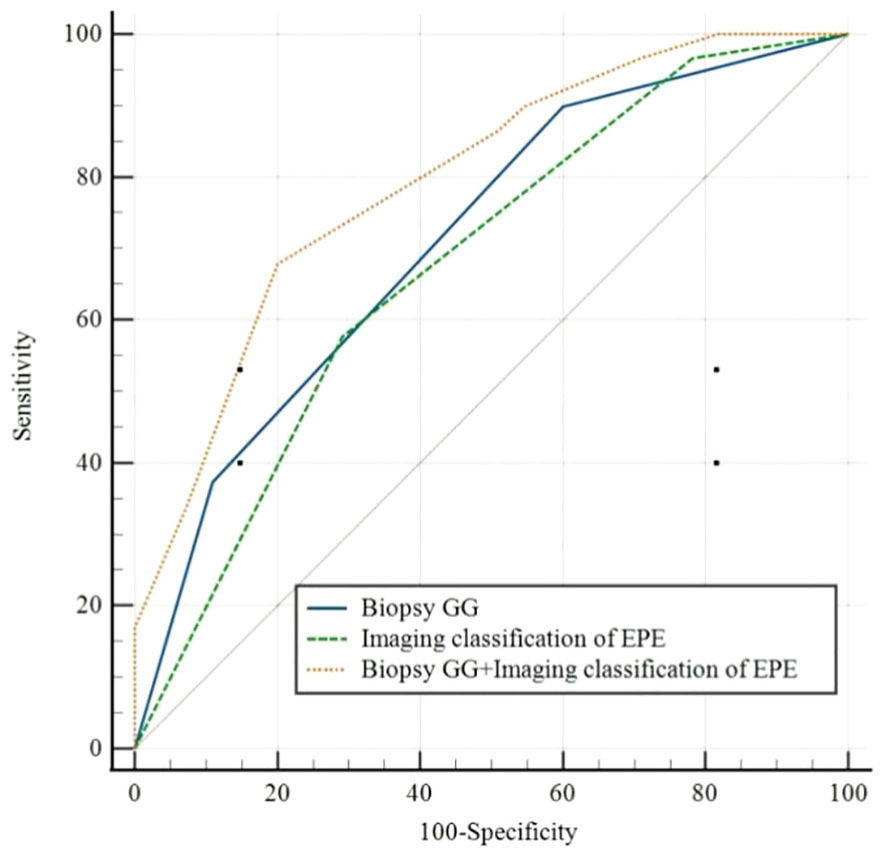
Receiver operating characteristic curves forBiopsy GG, Imaging classification of EPE, and Biopsy GG+ Imaging classification of EPE. EPE, extraprostatic extension; GG, grade group.

According to the ROC curve analysis of PSM, the AUC of biopsy GG and EPE imaging classification were, respectively, 0.712 (95% CI 0.620-0.793, P<0.05) and 0.677 (95% CI 0.583-0.7617, P<0.05), while the AUC of the combination of risk factors was 0.795 (95% CI 0.709-0.865, P<0.05) (**Figure 1**; **Table 4**). Particularly, the difference in the predictive value of biopsy GG and EPE imaging classification for PSM was not statistically significant (P>0.05). Nonetheless, when these indicators were combined, a significantly higher predictive performance was noted compared to that of any single indicator (P<0.05) (**Table 5**).

**Table 4:** ROC analysis for positive surgical margin.

	AUC	95%CI	P-value
Biopsy GG	0.712	0.620-0.793	<0.05
Imaging classification of EPE	0.677	0.583-0.761	<0.05
Biopsy GG+ Imaging classification of EPE	0.795	0.709-0.865	<0.05

AUC, area under curve; CI, confidence interval; EPE, extraprostatic extension; GG, grade group.

**Table 5:** Comparison of AUC value in different positive surgical margin prediction schemes.

	Biopsy GG	Imaging classification of EPE	Biopsy GG+ Imaging classification of EPE
Biopsy GG			
Imagingclassification of EPE	0.610		
Biopsy GG+ Imaging classification of EPE	<0.05	<0.05	

AUC, area under curve; EPE, extraprostatic extension; GG, grade group.

### 3.5 Clinical decision curves of the extracapsular extensions imaging classification, biopsy grade groups, and combined model

**Figure 2:**
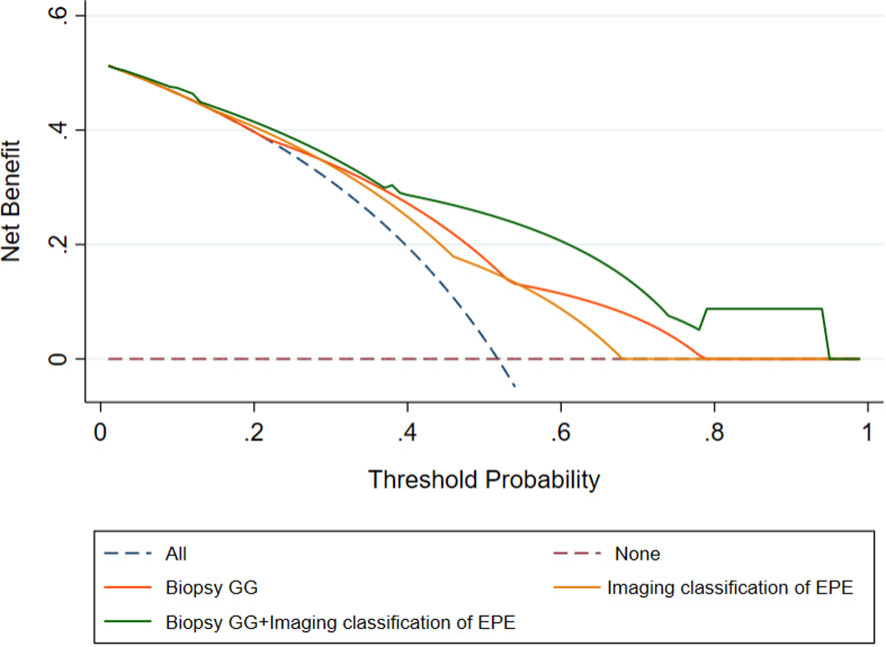
Decision curves of the Biopsy GG, Imaging classification of EPE, and Biopsy GG+ Imaging classification of EPE model for diagnosing positive surgical margin. EPE, extraprostatic extension; GG, grade group.

The analysis of clinical decision curves under different risk thresholds, the locations of curves for predicting PSM based on biopsy GG, image classification of EPE, and the combined model are in the upper right corner of the two extreme curves, indicating higher net benefits of these factors. Under most risk thresholds, the net benefit of the combined model was significantly higher than that of biopsy GG and EPE image classification (**Figure 2**).

### 3.6 Probability of positive surgical margins stratified by the image classification of extracapsular extensions and the biopsy grade groups

According to the probability of PSM stratified in terms of biopsy GG (1-2 vs. 3-4 vs. 5) and image classification of EPE (Type I vs. Type II vs. Type III), the combined prediction of PSM post-LRP is described herein. When the biopsy GG is 1-2, irrespective of the image classification of EPE, the risk of PSM was low (21%, 6/29). In the case of Type I image classification of EPE, irrespective of the biopsy GG, the risk of positive surgical margins was also low (14%, 2/14). The risk of PSM was higher when the EPE image classification was Type III combined with biopsy GG 3-5 (78%-90%) or biopsy GG 5 combined with image classification of EPE Type II (79%), in contrast to biopsy GG 1-2 (0%-31%) or Type I image classification of EPE (0%-50%) (**Table 6**).

**Table 6:** Probability of positivesurgical margin stratified with biopsy GGand Imaging classification of EPE.

Biopsy GG		1-2	3-4	5	Total
Imaging classification of EPE	Type I	0/7 (0%)	0/3 (0%)	2/4 (50%)	2/14 (14%)
	Type II	2/9 (22%)	9/27 (33%)	11/14 (79%)	22/50 (44%)
	Type III	4/13 (31%)	21/27 (78%)	9/10 (90%)	34/50 (68%)
Total		6/29 (21%)	30/57 (53%)	22/28 (79%)	58/114 (51%)

Date are shown by” the number of cases with surgical margin(+)/total case” and the percentage. The white, blue, and orange areas indicate the low (<40%), intermediate (40-60) and high(>60%) probability of positive surgical margin. EPE, extraprostatic extension; GG, grade group.

## 4 Discussion

Preoperative mp-MRI and clinical pathological data were used to evaluate the risk of PSM in 114 patients with PT3a stage PCa who had undergone LRP. EPE imaging classification and biopsy GG of PCa were found as risk factors for PSM. Combining these risk factors can potentially improve the predictive accuracy of PSM in LRP.

Mp-MRI has a high specificity but low sensitivity in predicting PSM. The establishment of a model based on mp-MRI to predict PSM in prostate cancer, revealed that tumor contact with the capsule length was a risk factor for PSM (17). MRI-based EPE score is significantly correlated with PSM (18). Establishment of a clinical prediction model for PSM revealed that the prostate imaging-reporting and data System (PI-RADS) score based on mp-MRI is a risk factor for PSM (19); the higher the PI-RADS score, the greater the likelihood of PSM (20). Over recent years, imaging genomics has developed rapidly; accordingly, the AUC of the imaging genomics model based on mp-MRI for the prediction of PSM was 0.78 (21). Preoperative mp-MRI was reported to be of moderate diagnostic efficacy in predicting PSM in the PT3a stage of PCa, with an AUC of 0.63 (22), which is lower than that of this study (0.67).Previous studies did not consider that the site of EPE in prostate cancer would affect PSM after LRP, this study proposed a new perspective, classifying and refining the EPE according to the anatomic site of EPE, which improved the predictive accuracy of PSM in LRP. In the multivariate regression analysis of postoperative PSM in our cohort of PT3a cases, imaging classification of EPE was found to be a risk factor for PSM in LRP for pT3a stage PCa.

The anatomical structures around the prostate may vary; hence, the extent of outward expansion of PCa varies in certain specific locations. The posterior and lateral parts of the prostate are relatively protected by nearby fascial structures, that slow down the process of tumor invasion (23). Therefore, the rate of PSM is higher in anterior PCa than in posterior and lateral PCa (3). The incidence of PSM in patients with tumors in the transition zone in the anterior part is significantly higher than in patients with tumors in the peripheral zone (P<0.01) (24). Our PT3a cases had a PSM rate of 50.8%. The PSM for EPE imaging subtypes I, II, and III were 14.2%, 44.0%, and 68.0%, respectively. These groups exhibited statistically significant differences. These results are related to the anatomical characteristics of different EPE imaging subtypes. Type I EPE does not involve the neurovascular bundles (NVBs) and seminal vesicles and has an intact capsule. Standard LRP procedure generally does not increase the risk of EPE-related PSM. Type II EPE is more likely to involve the posterolateral NVBs. Considering postoperative sexual and urinary functions, urologists attempt to preserve the nerves and blood vessels in the posterolateral aspect of the prostate, causing an increase in the rate of PSM. Type III EPE characterizes the fusion of the anterior capsule with the anterior fibromuscular stroma. Anterior prostate cancer can easily invade the anterior fibromuscular stroma, and its visibility is obstructed by the venous complex and puboprostatic ligament. Therefore, theoretically, patients with type III EPE have a significantly higher risk of PSM. These patients need to strictly adhere to surgical indications, and the risks of PSM should be lucidly explained to the patients and their families.

Clinical pathological factors are important in the prediction of PSM in LRP; they have shown high accuracy in previous studies. PSA level is a predictive factor for PSM (25), when PSA level exceeds 10 ng/mL, the risk of PSM increases significantly (5). The positive biopsy core percentage indirectly reflects tumor volume and burden; the larger the percentage of positive biopsy cores, the larger the tumor, and the higher the risk of PSM (26). Hens et al. showed that with the increase in Gleason score of biopsy GG, the risk of PSM also increases (27). In this study, PSA level, percentage of positive biopsy cores, and biopsy GG were significantly different between PSM and NSM. Among these, biopsy GG was identified as a risk factor for positive margins.

Combining mP-MRI and clinical pathological factors can improve the accuracy of PSM prediction in LRP. In this study, the AUC for predicting PSM using EPE imaging subtypes was 0.677. Combining EPE imaging subtypes with biopsy GG could increase the AUC to 0.795, with statistically significant differences. Patients with EPE imaging subtype III and biopsy GG 3-5, had the PSM rate of 81% (30/37). Patients with biopsy GG 5 and EPE imaging subtypes II-III had the PSM rate of 83% (20/24, while patients with EPE imaging subtypes I-II and biopsy GG 1-4 had a positive margin rate of 24% (11/46).

However, this study had certain limitations. First, it was a retrospective study with a small sample size, and the surgeries were performed by multiple urologists, which may lead to potential selection bias, Studies have shown that the proficiency score was used to assess the quality of urological surgery early, and the more experienced urological surgeons, the positive of surgical margins was lower (28). Second, the grouping of EPE imaging subtypes involved subjective judgment and may thus have grouping bias.

## 5 Conclusion

The PSM of PCa in stage PT3a was independently associated with EPE imaging classification and biopsy GG. Assessment of these factors comprehensively helps predict the probability of preoperative PSM. This assists urologists in deciding the preservation of the dorsal vein complex and NVBs during LRP.

## Data availability statement

The original contributions presented in the study are included in the article/supplementary material. Further inquiries can be directed to the corresponding author.

## Ethics statement

The studies involving humans were approved by Ningbo Yinzhou No. 2 Hospital Medical Ethics Committee. The studies were conducted in accordance with the local legislation and institutional requirements. The ethics committee/institutional review board waived the requirement of written informed consent for participation from the participants or the participants’ legal guardians/next of kin because Our ethics Committee supported our retrospective study without the need for informed consent. Written informed consent was not obtained from the individual(s) for the publication of any potentially identifiable images or data included in this article because Our ethics Committee supported our retrospective study without the need for informed consent.

## Author contributions

J-GW: Writing – original draft. CZ: Writing – review & editing. K-CZ: Writing – review & editing. J-BC: Writing – review & editing.
